# Early detection of immunoglobulin A against a vaccine backbone protein enables fast, durable, and objective identification of individuals vaccinated against Ebola virus

**DOI:** 10.1016/j.ebiom.2026.106515

**Published:** 2026-10-16

**Authors:** Olivia Posadas, Irina Maljkovic Berry, Viviane Callier, Stephen B. Kennedy, Mark Kieh, Sarah M. Browne, Olivier Tshiani Mbaya, Krystle Hensley, Lisa E. Hensley, Jens H. Kuhn, Ian Crozier, Richard T. Davey, H. Clifford Lane, Dean A. Follmann, Surender Khurana

**Affiliations:** a Division of Viral Products, Center for Biologics Evaluation and Research, U.S. Food and Drug Administration, Silver Spring, MD, 20993, USA; b Integrated Research Facility at Fort Detrick, Division of Clinical Research, National Institute of Allergy and Infectious Diseases, National Institutes of Health, Fort Detrick, Frederick, MD 21702, USA; c Clinical Monitoring Research Program Directorate, Frederick National Laboratory for Cancer Research, Frederick, MD, 21702, USA; d Partnership for Research on Vaccines and Infectious Diseases in Liberia, Monrovia, 1000, Liberia; e Laboratory of Immunoregulation, National Institute of Allergy and Infectious Diseases, National Institutes of Health, Bethesda, MD, 20892, USA; f Biostatistics Research Branch, National Institute of Allergy and Infectious Diseases, National Institutes of Health, Bethesda, MD, 20892, USA; g Institut National de Recherche Biomédicale, Ministry of Public Health, Kinshasa, Gombe, 12000, Democratic Republic of the Congo

**Keywords:** Ebola virus, Vaccine, Antibody, VSV, Vaccination, Diagnostics, Serodiagnosis, IgA, Outbreak response, Bundibugyo, Viral hemorrhagic fever, Multiplex assay, Surveillance

## Abstract

**Background:**

rVSVΔG-ZEBOV-GP vaccine is recommended for prevention of Ebola virus (EBOV) infection and disease (EVD). However, breakthrough EBOV infections have been observed in vaccinees. Currently available diagnostic and research assays cannot reliably distinguish individuals vaccinated against Ebola virus from those previously infected with the virus. This limitation complicates interpretation of vaccine-effectiveness studies, seroepidemiologic investigations, and estimates of infection or attack rates. The ability to differentiate vaccine-induced immune responses from those resulting from natural infection remains an important unmet public health and research need.

**Methods:**

We developed a multiplex sero-diagnostic assay simultaneously detecting IgA, IgG, and IgM antibodies against vesicular stomatitis Indiana virus nucleoprotein (VSV–N), a vaccine-specific marker absent in natural infection, and EBOV-GP, the vaccine's target antigen. We evaluated longitudinal samples from 30 individuals after vaccination and 30 placebo (saline) recipients, as well as 8 participants from a long-term vaccination follow-up study.

**Findings:**

We detected increase in all antibodies against both protein antigens in vaccinated individuals. Critically, the assay reveals that anti-VSV-N IgA responses emerge earlier and more robustly than anti-GP responses in vaccinees, establishing anti-N IgA as a definitive, early, and durable biomarker of rVSVΔG-ZEBOV-GP vaccination with high sensitivity and specificity.

**Interpretation:**

The assay enables rapid, accurate identification of vaccine recipients and supports real-time assessment of vaccine effectiveness during outbreaks. Furthermore, the assay's adaptability to the expanding pipeline of VSV-vectored vaccines positions it as a transformative diagnostic platform for accelerating preparedness for future infectious disease threats and outbreak response strategies.

**Funding:**

Center for Biologics Evaluation and Research (CBER) at the 10.13039/100000038U.S. Food and Drug Administration (FDA). The 10.13039/100000054National Cancer Institute (NCI) and the Divisions of Intramural Research (DIR) and Clinical Research (DCR) of the 10.13039/100000060National Institute of Allergy and Infectious Diseases (NIAID) at the U.S. 10.13039/100000002National Institutes of Health (NIH).

**Research in context**

**Evidence before this study**

We searched PubMed and Embase from inception through May 2026, without language restrictions, using the terms “rVSV-ZEBOV”, “Ebola vaccine”, “vesicular stomatitis virus”, “sero-diagnostic”, “vaccine-induced antibody”, “IgA”, “IgG”, “IgM”, and “vaccination status”. Existing sero-diagnostic assays for EVD predominantly target anti-EBOV glycoprotein IgG and cannot reliably distinguish vaccinated-but-uninfected individuals from those with natural infection or vaccinated with breakthrough infection, a critical limitation in increasingly vaccinated populations. The primary limitations of serodiagnosis, such as delayed detection, cross-reactivity, and the inability to distinguish between active infection, past exposure, and vaccination, make differentiating test results clinically important for guiding accurate patient treatment, enforcing proper quarantine measures, and reliably tracking disease outbreaks. Small studies in US and European phase I trial participants found limited or transient anti-VSV humoural responses following rVSVΔG-ZEBOV-GP vaccination. More recent work in African vaccinees demonstrated that anti-VSV-N IgG can identify vaccinated individuals and correlate with anti-EBOV-GP IgG, but these studies relied on single-isotype and did not evaluate early post-vaccination timepoints or IgA responses.

**Added value of this study**

We describe a multiplex assay simultaneously measuring IgM, IgG, and IgA antibodies against both EBOV-GP and VSV-N. We identified an unexpected early, high-magnitude, and durable anti-VSV-N IgA response as a highly sensitive (96.7%) and specific (93.3%) biomarker of rVSVΔG-ZEBOV-GP vaccination detectable as early as day 7, well before anti-GP antibody responses are detected, and persistent for at least 1 year. This finding was observed in two independent vaccinated cohorts and across multiple pragmatically relevant sample-handling conditions.

**Implications of all the available evidence**

Accurate, early serological identification of rVSVΔG-ZEBOV-GP vaccine recipients is important for evaluating real-world vaccine effectiveness and characterising breakthrough infections. Anti-VSV-N IgA detection addresses an unmet differential diagnostic need, enabling accurate identification of rVSVΔG-ZEBOV-GP vaccinated individuals, allowing for the detection of breakthrough infections in vaccinated populations, facilitating epidemiological tracking and the evaluation of vaccination campaign effectiveness in endemic and outbreak-affected regions. This assay framework is adaptable to the growing pipeline of rVSV-vectored vaccines, including for the prevention of Bundibugyo virus, Sudan virus, and Lassa virus infections, strengthening serological surveillance infrastructure for future outbreak responses.

## Introduction

Ebola virus disease (EVD) is a severe disease caused by Ebola virus (EBOV; family *Filoviridae*: species *Orthoebolavirus zairense*^1^) that is associated with an average case fatality rate (CFR) of ≈44%. Since 1976, 25 EVD outbreaks have been reported primarily in sub-Saharan Africa, causing ≈34,000 cases that resulted in ≈15,000 deaths.^2^ Demonstration of clinical efficacy (in 2013–16 in Western Africa)^3^ and subsequent real-world effectiveness (in 2018–20 in the Democratic Republic of the Congo)^4^ of the rVSVΔG-ZEBOV-GP vaccine established rVSVΔG-ZEBOV-GP as a valuable addition to the EVD outbreak and preventative management toolkit to reduce EVD-associated deaths and morbidity. In contrast, in the context of the 2026 outbreak of Bundibugyo virus disease (BVD), for which no licenced vaccines are available, rapidly increasing case numbers and deaths (8728 case numbers and 4205 deaths, as of October 6, 2026)^5^ are stark reminders of the challenges in the management of filovirus disease outbreaks without an approved vaccine. The rVSVΔG-ZEBOV-GP (Ervebo) encodes the glycoprotein (GP) of EBOV variant Kikwit). The 2026 Bundibugyo virus outbreak is caused by Bundibugyo virus (BDBV; species *Orthoebolavirus bundibugyoense*), which differs from EBOV at the nucleotide level by approximately 30%. Given the evolutionary divergence between EBOV and BDBV GP immunogens, it is unclear whether rVSVΔG-ZEBOV-GP can provide cross-protection against BVD; prior cross-protection studies in nonhuman primates have shown limited or no protection against BDBV challenge following rVSVΔG-ZEBOV-GP vaccination,^6^ and no efficacy data in humans are currently available.

As the EBOV GP binds to the host-cell receptor to enable viral particle entry into the cell, it is the main target for development of vaccines and monoclonal antibody (mAb)-based medical countermeasures for EBOV/EVD.^7^^,^^8^ Of the two EBOV/EVD vaccines that are recommended for general use by the WHO against EBOV, only the rVSVΔG-ZEBOV-GP vaccine is licenced by both the U.S. FDA and the European Medicines Authority and is used during EVD outbreaks. rVSVΔG-ZEBOV-GP is a replication-competent, attenuated recombinant vesicular stomatitis Indian virus (VSV) vector backbone expressing EBOV GP.^9^ Notwithstanding the obvious value, both in ring vaccination (of contacts and their close contacts in active outbreaks) and preventive (in at-risk health care workers) vaccine strategies, questions persist regarding the precise efficacy and durability of the rVSVΔG-ZEBOV-GP vaccine. EBOV infections have been recorded within ten days of vaccine receipt, likely due to insufficient time between vaccination and exposure for effective immune responses to develop (https://www.cdc.gov/acip/grade/ebola-vaccine.html). In addition, true “breakthrough” infections (after vaccine receipt) are well-documented: for example, of EBOV RT-PCR-confirmed patients, 38/155 (24.5%)^10^ and 41/175 (23.4%)^11^ reported vaccination more than ten days prior to enrolment in a randomised controlled trial or admission to a treatment centre, respectively. Available evidence suggests that these infections may be associated with less severe disease and reduced CFR^4^^,^^12^; nonetheless, reports of fatal EVD and the continuation of ongoing human-to-human transmission chains after vaccine breakthrough infections underscore the devastating individual and public health consequences of these events.^13^ Finally, the true durability of *effective* vaccine-mediated protection is unknown, and open questions remain about the need and best strategies for booster vaccine for prevention against EVD.^14^^,^^15^

Effectively addressing these questions in increasingly vaccinated populations (that include EVD survivors), requires accurate identification of previously rVSVΔG-ZEBOV-GP-vaccinated individuals. Historically, patient self-report of vaccination status has been the only data available, and self-reported prior vaccination has been associated with improved clinical outcomes and milder disease in patients admitted to treatment units.^16^ However, vaccination status cannot always be accurately determined, particularly in cases involving incapacitated patients, or when vaccination records verifying status and vaccination dates are unavailable or incomplete.^12^ Furthermore, current EBOV-GP-based sero-diagnostic assays cannot reliably differentiate between vaccine-induced and infection-induced immunity because they detect IgG antibodies to EBOV-GP–whether those derive from infection or rVSVΔG-ZEBOV-GP immunisation.^10^^,^^17^^,^^18^ Thus, development of assays that can reliably distinguish between vaccination-induced and infection-induced immunity would address a critical gap by enabling objective confirmation of vaccination status. This would allow for more accurate estimation of vaccine effectiveness (e.g., in “test-negative” observational studies) and epidemiological investigations, and improve public health decision making.

We previously described “Ebola-Detect”, a highly specific and sensitive enzyme-linked immunosorbent assay (ELISA) for detection of EBOV infection-induced antibodies against various EBOV proteins not encoded by rVSVΔG-ZEBOV-GP.^19^ This ELISA did not include VSV antigens to determine vaccine-induced antibodies. Recently, the utility of a multi-antigen IgG assay to confirm vaccination status was demonstrated; however, the early kinetics of the humoural responses, and the role of alternative vaccine-induced antibody isotypes (e.g., IgA) have not been fully characterised.^20^ A more granular understanding of the kinetics and magnitude of both vector-induced (anti-VSV) and antigen-induced (anti-EBOV-GP) antibody responses following rVSVΔG-ZEBOV-GP vaccination, including the detection of IgM and IgA, would improve robust identification of vaccination status and understanding dynamics of vaccine vector immunogenicity. It should be noted that anti-VSV-N antibody levels reflect immunogenicity of the vaccine backbone but do not serve as direct indicators of vaccine-mediated protection against EBOV. Therefore, we developed a multiplex serodiagnosis assay (termed Ebola-Detect “2.0”) that simultaneously detects IgM, IgG, and IgA antibodies to the VSV vaccine backbone and EBOV-GP. Through analysis of two rVSVΔG-ZEBOV-GP-vaccinated cohorts from Liberia and the US, we demonstrate that an unexpectedly early and robust IgA response against the VSV nucleoprotein (N) is a highly sensitive and specific biomarker of rVSV-based vaccine receipt.

## Methods

### Human samples

Two sets of samples were used for analysis. The first set consisted of longitudinal serum samples from 60 participants enrolled in the Partnership for Research on Ebola Virus in Liberia (PREVAIL) 1 study^21^ (Fig. 1). The PREVAIL 1 study was a randomised placebo-controlled, double-blinded clinical trial of preventive vaccines conducted in Liberia during the 2013–2016 Western African EVD outbreak. PREVAIL 1 enrolled volunteers without a history of EVD that had either received the rVSVΔG-ZEBOV-GP, chimpanzee adenovirus 3 (ChAd3-EBO Z)-based vaccine, or a saline placebo (that did not include the rVSV backbone). We performed a retrospective analysis of longitudinal samples collected on days 0, 7, 28, 180, and 360 post-vaccination from 30 randomly selected rVSVΔG-ZEBOV-GP-vaccinated adults, and 30 randomly selected control group samples collected on days 0 and 28 after saline placebo vaccine administration. In this retrospective analysis of randomly selected samples from the PREVAIL 1 cohort, no formal sample size calculation was performed. Few rVSVΔG-ZEBOV-GP-vaccinated adults (*n* = 4) were also sampled on days 3, 10, and 14 (Fig. 1). Table 1 details the baseline characteristics of the PREVAIL cohort.

**Fig. 1:**
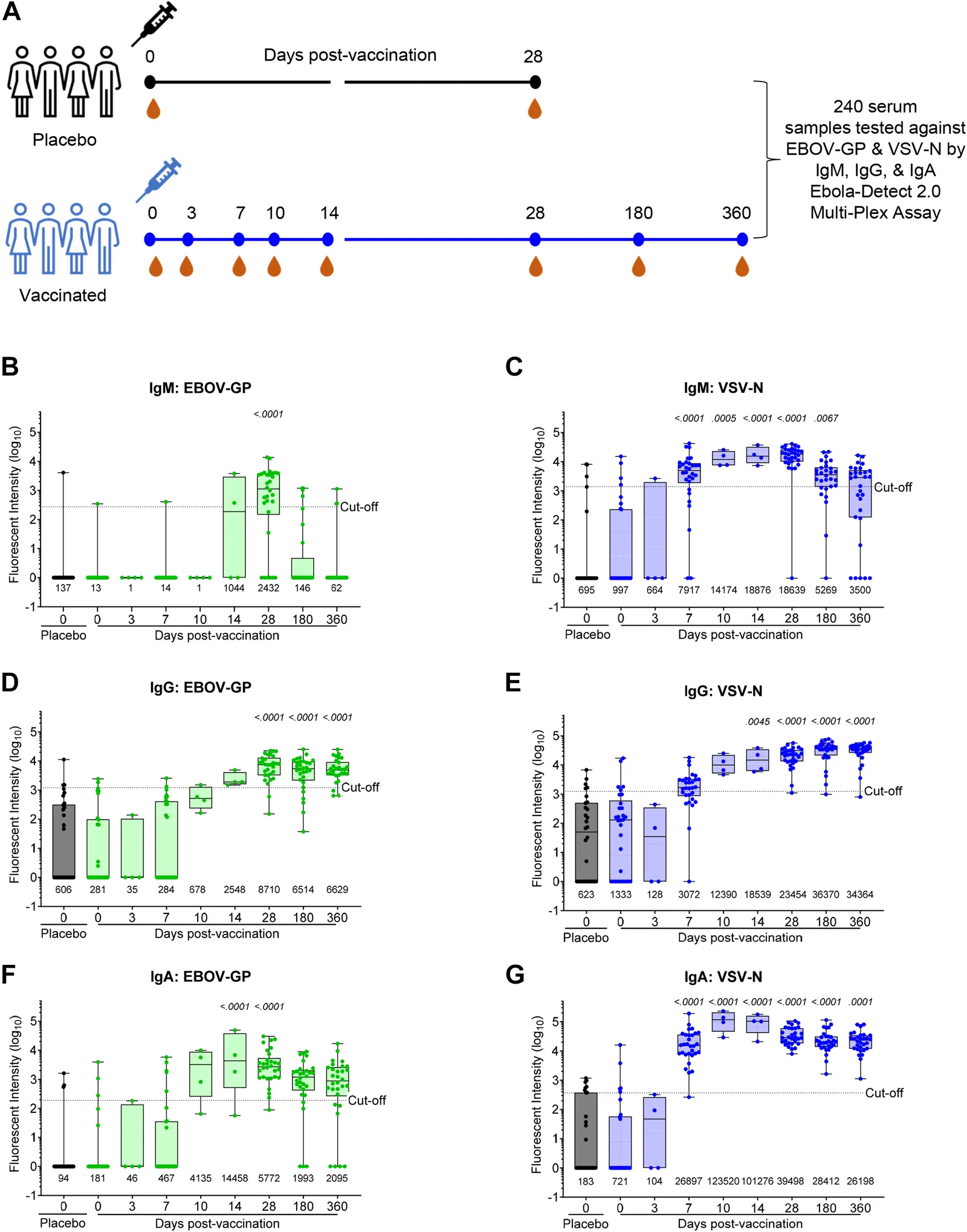
**rVSVΔG-ZEBOV-GP vaccination results in higher antibody responses to vaccine backbone antigen compared with anti-vaccine target antigen**. (A) Design of the longitudinal rVSVΔG-ZEBOV-GP vaccine study. (B–G) Box and whisker plots of fluorescent intensity indicating antibody levels at a 100-fold serum dilution for longitudinal samples collected on days 0, 7, 28, 180, and 360 post-vaccination from 30 randomly selected rVSVΔG-ZEBOV-GP-vaccinated adults (*n* = 30 for days 0, 7, 28, 180, and 360; *n* = 4 for days 3, 10, and 14), and 30 randomly selected control group samples collected on day 0 after saline placebo vaccine administration. against Ebola virus (EBOV) glycoprotein (GP) and vesicular stomatitis Indiana virus (VSV) nucleoprotein (N) by IgM (B and C), IgG (D and E) and IgA (F and G). The means of fluorescent intensity values for each timepoint are listed below the plotted data. Placebo group day 0 values serve as a reference (saline recipients), not as the pre-vaccination baseline of the rVSVΔG-ZEBOV-GP-vaccinated cohort. All statistical comparisons were performed between vaccinated individuals at each post-vaccination timepoint and the day 0 placebo values using one-way ANOVA with mixed-effects analysis. A False Discovery Rate (FDR) adjustment (Benjamini-Hochberg procedure) was applied to correct for multiple testing and resulting significant *p*-values (<0.05) are shown above each timepoint.

**Table 1:** Demographics of PREVAIL 1 cohort.

Group	Placebo (n = 30)^a^	Vaccine n = (30)^a^
Age at enrolment (years)	32 (10)	31 (10)
Sex		
Female	12 (40%)	16 (53%)
Male	18 (60%)	14 (47%)
BMI	23.0 (6.2)	22.5 (4.0)
HIV	3 (10%)	1 (3.4%)
Syphilis status: VISIT 0	2 (6.7%)	3 (10%)

a Mean (SD); n (%).

The second analysis set consisted of whole blood samples that were collected from eight adults who had been vaccinated with rVSVΔG-ZEBOV-GP five to six years previously, as part of an open-label phase 2 randomised controlled trial characterising the effects of immunisation with rVSVΔG-ZEBOV-GP in healthy adults in the United States (PREPARE).^14^ To assess the impact of inactivation/sample-handling and sample freeze-thaws on antibody detection, these whole blood samples were divided into aliquots and each aliquot was treated according to six sample-handling conditions: 1) whole blood alone (WB); 2) whole blood that was freeze-thawed (FT) (frozen at −80 °C and thawed at room temperature for 10 min) four times; 3) gamma-irradiated whole blood with 5 Mrad (IRR); 4) whole blood FT and IRR; 5) whole blood treated with 2% sodium dodecyl sulphate (SDS) and heat-inactivated (HI) at 96 °C for 5 min; and 6) whole blood FT, HI, and SDS. A matched contemporaneous serum sample was also extracted for comparative analysis.

All collected samples were analysed; demographic data regarding sex were not analysed separately in this study. Both male and female participants were included, and sex was not considered as a biological variable in this study. No formal sample size calculation was performed for this retrospective study.

### Study approval

Ethical approval for PREVAIL 1 (ClinicalTrials.gov ID: NCT02344407) was obtained from the National Research Ethics Board of Liberia and the NIAID Institutional Review Board (IRB) at the National Institutes of Health. Whole blood samples were also obtained from eight participants enrolled in an NIH Clinical Research Center IRB-approved normal volunteer blood donation protocol. Specimens were collected only from individuals who had consented (or assented) in writing to collection, storage, and future use of these samples for research purposes.

The study at the Center for Biologics Evaluation and Research (CBER), FDA, was conducted with de-identified samples under Research Involving Human Subjects (RIHSC) exemption #15-0B; and all assays performed fell within the permissible usages in the original consent.

### Ebola-detect 2.0 multiplex assay

We developed a fluorescent magnetic bead multiplex immunoassay consisting of uniquely identifiable microspheres (BioRad Cat # MC10034, Cat # MC10042,and Cat # MC10053) that were conjugated using amine-coupling kit (BioRad Cat # 171406001) to either purified recombinant EBOV variant Kikwit GP (in-house; homotypic to the Kikwit GP encoded by rVSVΔG-ZEBOV-GP), VSV-N (in-house), or control human serum albumin (HSA), using a Bio-Plex amine coupling kit (BioRad). Purified recombinant EBOV variant Kikwit GP was produced in HEK293 cells, as described previously.^22^ The his-tagged VSV-N protein was obtained from supernatant of *E. coli* cell lysate and purified using the Nickel column followed by gel-filtration chromatography with Superdex 200 column and stored at −80 °C in PBS, 500 mM NaCl, 10% Glycerol, pH 7.4. Following confirmation of coupling, the multiplex immunoassay was performed on the samples as described previously.^23^ Briefly, equal number of beads for each unique protein-conjugated microsphere were added in a tube and mixed. Whole blood samples were thawed and immediately precipitated at 7000×*g* for 1 min, sera were collected, diluted to 1:50 in assay buffer (20 mM PBS pH 7.2 containing 1% BSA and 0.05% Tween-20), and added to a dilution plate. Equal volumes of bead-mix and serum dilutions were combined, resulting in final serum concentrations of 100-fold dilution in 96-well plates. Following incubation at room temperature for 2 h, wells were washed with Bio-Plex Pro wash buffer (BioRad Cat # 171304500) using a Bio-Rad Bio-Plex Pro II Wash Station (BioRad Cat # 30034376). A fluorophore-labelled secondary [Donkey anti-human IgG-Fc specific antibody (Cat # 709-005-098), Donkey anti-human IgM Fcμ specific (Cat # 709-005-073), goat anti-human serum IgA alpha chain specific (Cat # 109-005-011) were purchased from Jackson Immuno Research and labelled with fluorophore as per manufacturer's instructions] antibody mix was added and incubated for 30 min at room temperature. The bead-containing wells were washed and then analysed with an LSR Fortessa X-20 (BD Biosciences). Fluorescent intensity was measured by FACSDiva software (BD Biosciences). Antibody levels (measured as mean fluorescent intensity, MFI) were determined. Fluorescent intensity data were normalised as follows: (1) blank background fluorescence (wells with beads and buffer but no serum) was subtracted from each sample; and (2) fluorescence intensity binding to control HSA beads was subtracted to account for non-specific background binding. For representation purposes, all negative values were set to zero. All experiments were performed twice, and the researcher performing the assay was blinded to sample identity.

For second analysis, whole blood samples were collected and aliquoted from eight previously vaccinated individuals. A separate portion of each whole blood sample was allowed to clot, and serum was isolated. Six experimental conditions were tested to test the effects of irradiation, freeze thaw, SDS treatment and heat inactivation on whole blood. The eight individual samples representing the six experimental conditions were centrifuged to collect cell-free supernatant and tested in the multiplex assay.

### Statistical analysis

Duplicate measurements of antibody concentrations at the 1:100 dilution were averaged to give one value per person per antibody per timepoint. In Fig. 2, the geometric means (means of the log_10_-transformed values, transformed back to linear scale) are given. Statistical analysis on the log_10_-transformed values was performed using Student's *t*-test or nonparametric Kruskal–Wallis test using Prism version 10.0.02 (GraphPad) and R software version 4.5.0. Statistical comparisons between groups were conducted with an alpha level of 0.05. A False Discovery Rate (FDR) adjustment (Benjamini-Hochberg procedure) was applied to correct for multiple testing.

**Fig. 2:**
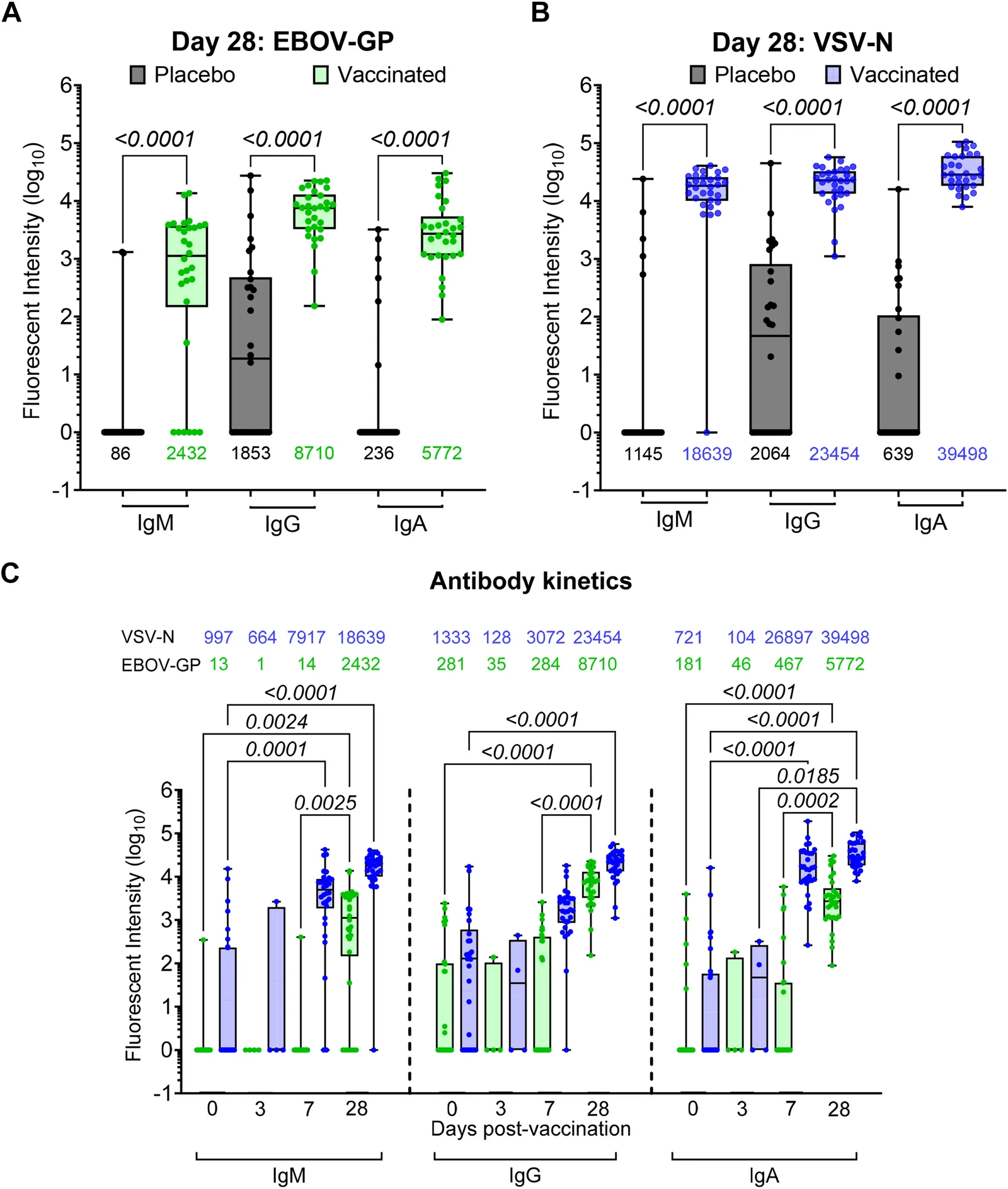
**rVSVΔG-ZEBOV-GP vaccination results in anti-vaccine backbone immunoglobulin A responses that precede anti-vaccine target antigen responses**. Fluorescent intensity of antibody isotype reactivity of 100-fold diluted samples from the saline placebo group (*n* = 30; in black) and the rVSVΔG-ZEBOV-GP-vaccinated group (*n* = 30; in colours) at day 28 post-vaccination against Ebola virus (EBOV) glycoprotein (GP) (A) and vesicular stomatitis Indiana virus (VSV) nucleoprotein (N) (B). (C) Temporal antibody kinetics following rVSVΔG-ZEBOV-GP vaccination. Comparison of IgM, IgG and IgA reactivity against VSV-N and EBOV-GP at days 0, 3, 7, and 28 following rVSVΔG-ZEBOV-GP vaccination (*n* = 30 for days 0, 7, and 28; *n* = 4 for day 3). Box and whisker plots, with interquartile ranges indicate the mean fluorescent intensity. Dots indicate the average values of the duplicates for each sample. Differences between groups were examined for statistical significance by one-way ANOVA and Kruskal–Wallis test, and only statistically significant values (*p*-values <0.05) are shown.

For sensitivity and specificity analysis, cut-off (threshold) MFI values were determined using receiver operating characteristic (ROC) analysis, which identifies the optimal value that maximises both sensitivity and specificity for distinguishing vaccinated individuals (days 7, 28, 180, and 360) from baseline pre-vaccination samples (day 0) of the rVSVΔG-ZEBOV-GP vaccinated group (true pre-vaccination baseline); day 0 samples from the saline placebo group served as an additional reference. Thresholds for sensitivity and specificity calculations were identified using the Youden Index to maximise both metrics. 95% confidence intervals (CIs) are reported. For whole-blood sample analyses, pairwise comparisons were performed between untreated whole blood and each sample-handling condition.

### Data and materials availability

All data are shown in the manuscript figures and the Figure S1. There are restrictions on the availability of the multiplex assay described in this study due to a US patent application. The assay technology can be made available on request from the corresponding author (S.K.) upon signing appropriate agreements, as required for patented invention.

### Ethics statement

The study at the Center for Biologics Evaluation and Research (CBER), FDA, was conducted with de-identified samples under Research Involving Human Subjects (RIHSC) exemption #15-0B; and all assays performed fell within the permissible usages in the original consent.

### Role of funders

The funders had no role in study design, data collection and analysis, decision to publish, or preparation of the manuscript.

## Results

### rVSVΔG-ZEBOV-GP vaccination results in higher antibody responses to vaccine backbone antigen compared with vaccine target antigen

Longitudinal antibody responses to EBOV-GP and VSV-N in vaccinated (*n* = 30) and placebo (*n* = 30) groups were evaluated in a blinded manner using the newly developed multiplex assay. IgA, IgG, and IgM responses were quantified on days 0, 3, 7, 10, 14, 28, 180, and 360 post-vaccination in the rVSVΔG-ZEBOV-GP group and on days 0 and 28 in the placebo group (Fig. 1A). Note that *n* = 30 applies to timepoints days 0, 7, 28, 180, and 360; and samples from four participants were collected as well at the intermediate timepoints (days 3, 10, and 14).

The multiplex assay demonstrated a clear immunological divergence between the two groups (Fig. 1B–G and Figure S1). Pre-vaccination (D0) binding antibodies were low and similar in both placebo and rVSVΔG-ZEBOV-GP vaccinated group against EBOV-GP and VSV-N. The rVSVΔG-ZEBOV-GP vaccinated individuals had rapidly increasing antibody levels to VSV-N that were detectable as early as day 7 post-vaccination. The kinetics of IgM, IgG, and IgA antibody isotype-specific responses to EBOV-GP and VSV-N varied. IgM responses against both EBOV-GP and VSV-N peaked around days 14–28 and then declined (Fig. 1B and C, and Figure S1). IgG and IgA responses against EBOV-GP increased gradually, peaking at days 14–28 [mean fluorescent intensity (MFI) ≈8710 and 14,458, respectively]; IgG responses against EBOV-GP remained elevated through day 360 (Fig. 1D, F and Figure S1).

The VSV-N-specific antibody levels were generally 5–10-fold higher compared with EBOV-GP-specific antibodies, possibly reflecting a greater immunogenic stimulus from the VSV nucleoprotein relative to the EBOV-GP insert within the rVSVΔG-ZEBOV-GP vector (Fig. 1B–G and Figure S1). VSV-N-specific responses were more robust, with IgG reaching an MFI of 23,454 by day 28 that continued to rise over time and was maintained at MFI = 34,364 at one-year post-vaccination (Fig. 1E). In individuals from the placebo group, antibody signals generally remained at or near background levels over 28 days, supporting the assay's specificity; however, a small number of participants in the placebo and vaccinated D0 groups showed signals above the cut-off value, which should be taken into account when interpreting the diagnostic thresholds (Figure S1). Notably, anti-VSV-N IgA appeared earlier and at higher reactivity than anti-GP antibodies at day 7 (MFI ≈26,897), with mean levels peaking at days 10–14 (MFI >100,000) and persisting until at least one year after vaccination (MFI ≈26,198) (Fig. 1G and Figure S1).

### rVSVΔG-ZEBOV-GP vaccination results in anti-vaccine backbone immunoglobulin A responses that precede anti-vaccine target antigen responses

Comparison of antibody responses at day 28 post-vaccination revealed higher IgM, IgG, and IgA levels against VSV-N and EBOV-GP in vaccinated individuals compared with saline placebo recipients (Fig. 2A and B). Antibody responses to EBOV-GP were lower but still increased post-vaccination, with MFI values of 2432, 8710, and 5772 for IgM, IgG, and IgA, respectively (Fig. 2A). For VSV-N, vaccinated individuals had MFI values of 18,639, 23,454, and 39,498 for IgM, IgG, and IgA, respectively, compared with near-baseline levels (MFI = 639–2064) at D28 in the placebo group (Fig. 2B). Overall, anti-VSV-N antibodies showed a greater intra-isotype induction than anti-EBOV-GP antibodies at each timepoint (Fig. 2C). IgA levels against VSV-N were 57-fold higher than the anti-EBOV GP levels on day 7. A substantial increase in antibody levels occurred from day 7 to day 28 for most of the isotype–antigen combinations.

### The multiplex assay identifies rVSVΔG-ZEBOV-GP vaccine-induced antibodies with high sensitivity and specificity as early as seven days post-vaccination

Receiver operating characteristic (ROC) analysis was used to evaluate the diagnostic performance of the multiplex assay (Fig. 3A–F). Sensitivity (Sen) and specificity (Spe) analyses were performed for each antibody–antigen pair for pre-vaccination baseline at days 0 versus post-vaccination antibody levels measured on days 7, 28, 180, and 360 in the rVSVΔG-ZEBOV-GP vaccinated group. Area under the curve (AUC) values approaching 1 indicated excellent diagnostic accuracy. IgG and IgA levels against EBOV-GP at day 28 were 93.3% sensitive and 93.3% specific at an MFI cutoff of 1401 and 299, respectively (Fig. 3B and C), whereas the sensitivity at day 7 post-vaccination was only 30–40%.

**Fig. 3:**
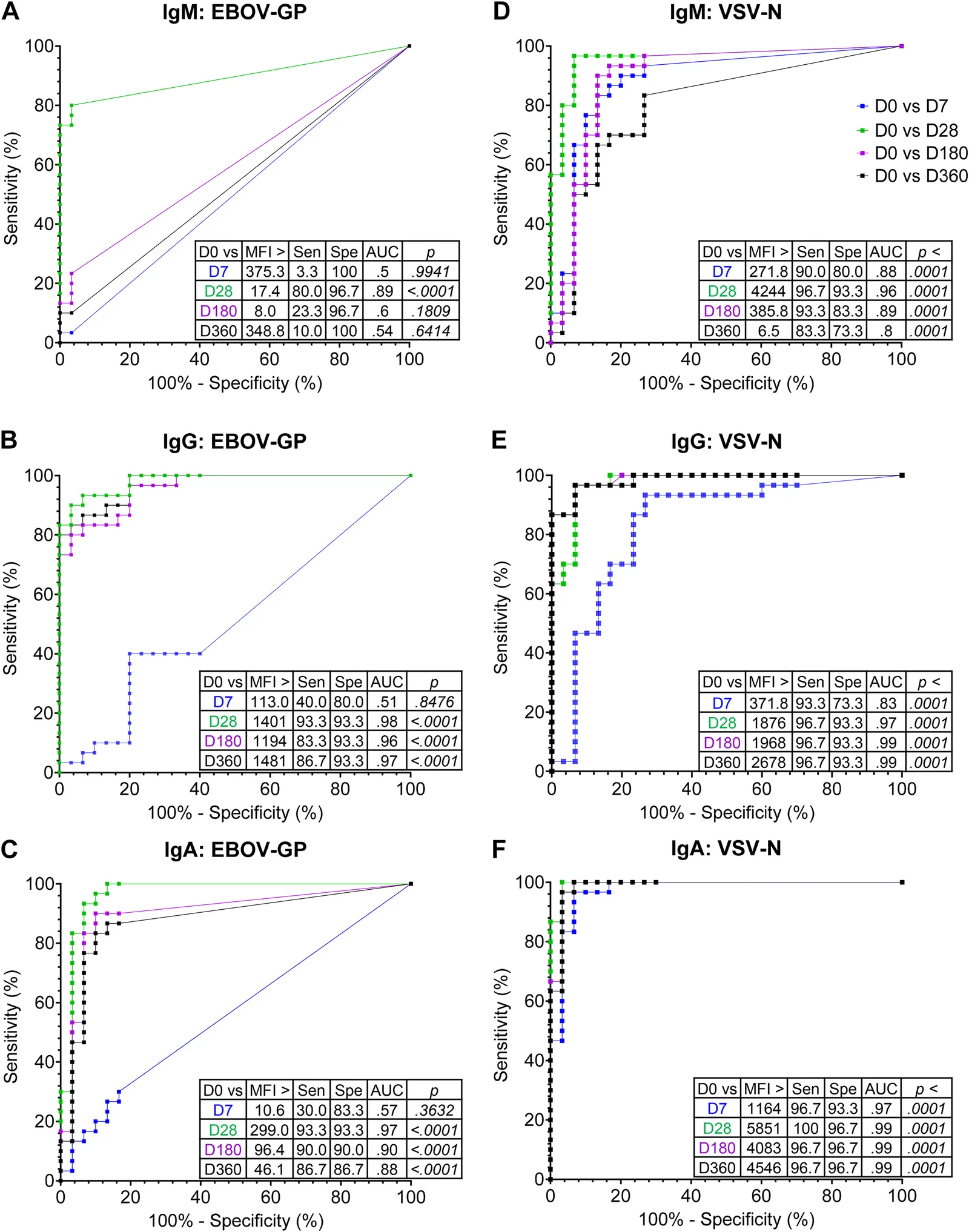
**The multiplex assay detects rVSVΔG-ZEBOV-GP vaccine-induced antibodies with high sensitivity and specificity as early as seven days post-vaccination**. Receiver operating characteristic (ROC) analysis for antibody reactivity of samples from 30 adults vaccinated with rVSVΔG-ZEBOV-GP. Area under the ROC analysis curve (AUC) was performed to calculate percent sensitivity (Sen) and specificity (Spe) values with 95% confidence intervals by comparing reactivity to Ebola virus (EBOV) glycoprotein (GP) (A–C) or vesicular stomatitis Indiana virus (VSV) nucleoprotein (N) (D–F) on baseline pre-vaccination (D0) samples versus post-vaccination antibody response on day 7 (D7; blue), day 28 (D28; green), day 180 (D180; purple), and day 360 (D360; black) for each time-point in the rVSVΔG-ZEBOV-GP-vaccinated group. Cut-off mean fluorescent intensity (MFI) values were determined separately for each time-point comparison by ROC analysis for IgM (A and D), IgG (B and E) and IgA (C and F) binding to EBOV-GP or VSV-N and are represented in the tables in the corresponding panels. Specimens with an absorbance greater than the cut-off value were considered seropositive and those with absorbance values lower than the cut-off value were considered seronegative. The variation for each sample in duplicate runs was <8%. The data shown are the average values of the two experimental runs. Supportive data for Figure 3 for the 95% CIs and standard error for sensitivity, specificity and AUC are shown in Table S1.

ROC analysis for anti-VSV-N antibody levels revealed >93% sensitivity and specificity from day 7 onwards, and both IgA and IgG maintained high serodiagnostic performance even at day 360, suggesting the assay's utility for both early and long-term immune monitoring (Fig. 3D, F). Anti-VSV-N IgA levels were the most accurate predictor of vaccination status (Fig. 3F) at day 28, ROC analysis indicated 100% sensitivity and 96.7% specificity at the threshold MFI >5851. As early as day 7, detection of anti-VSV-N IgA was 96.7% sensitive and 93.3% specific, i.e., was superior to the 73% and 80% specificity identified for IgG and IgM, respectively.

### Gamma irradiation with or without freeze-thawing is the optimal inactivation approach for detection of rVSVΔG-ZEBOV-GP vaccine-induced antibodies using the multiplex assay

To assess the potential reliability of the multiplex assay in the field with whole blood and under different storage or sample processing conditions, we next evaluated assay antibody detection between whole-blood and serum samples from rVSVΔG-ZEBOV-GP vaccinated individuals, and between multiple freeze-thaw and sample-handling combinations, to determine any impact of sample type and handling methods pragmatically relevant to samples available during an EVD outbreak (Fig. 4A). Untreated whole blood (WB) served as the primary comparator for all conditions, with contemporaneously extracted serum included as an additional reference. Whole blood samples from eight adults collected five to six years after rVSVΔG-ZEBOV-GP vaccination demonstrated that freeze-thaw (FT) and gamma irradiation (IRR) with or without FT preserved anti-VSV-N and anti-EBOV-GP antibody detection, with IRR and IRR + FT yielding comparable signals compared to untreated whole blood (and serum) for some isotypes (Fig. 4B–G). Sodium dodecyl sulphate (SDS)-based treatment of whole blood resulted in significant reduction in signal intensity with complete loss of binding for IgM and IgA antibodies. Following sample treatment, anti-EBOV-GP IgM antibodies were not detected, and anti-EBOV-GP IgA antibodies measurements were variable across handling conditions (Fig. 4B and D). Notably, the IRR and IRR + FT method performed well across all antibody isotypes against VSV-N with similar reactivity between whole blood and contemporaneously extracted sera, suggesting this inactivation approach is optimal for whole blood detection of vaccine-induced antibodies by the multiplex assay, even 5–6 years after rVSVΔG-ZEBOV-GP vaccination (Fig. 4E–G).

**Fig. 4:**
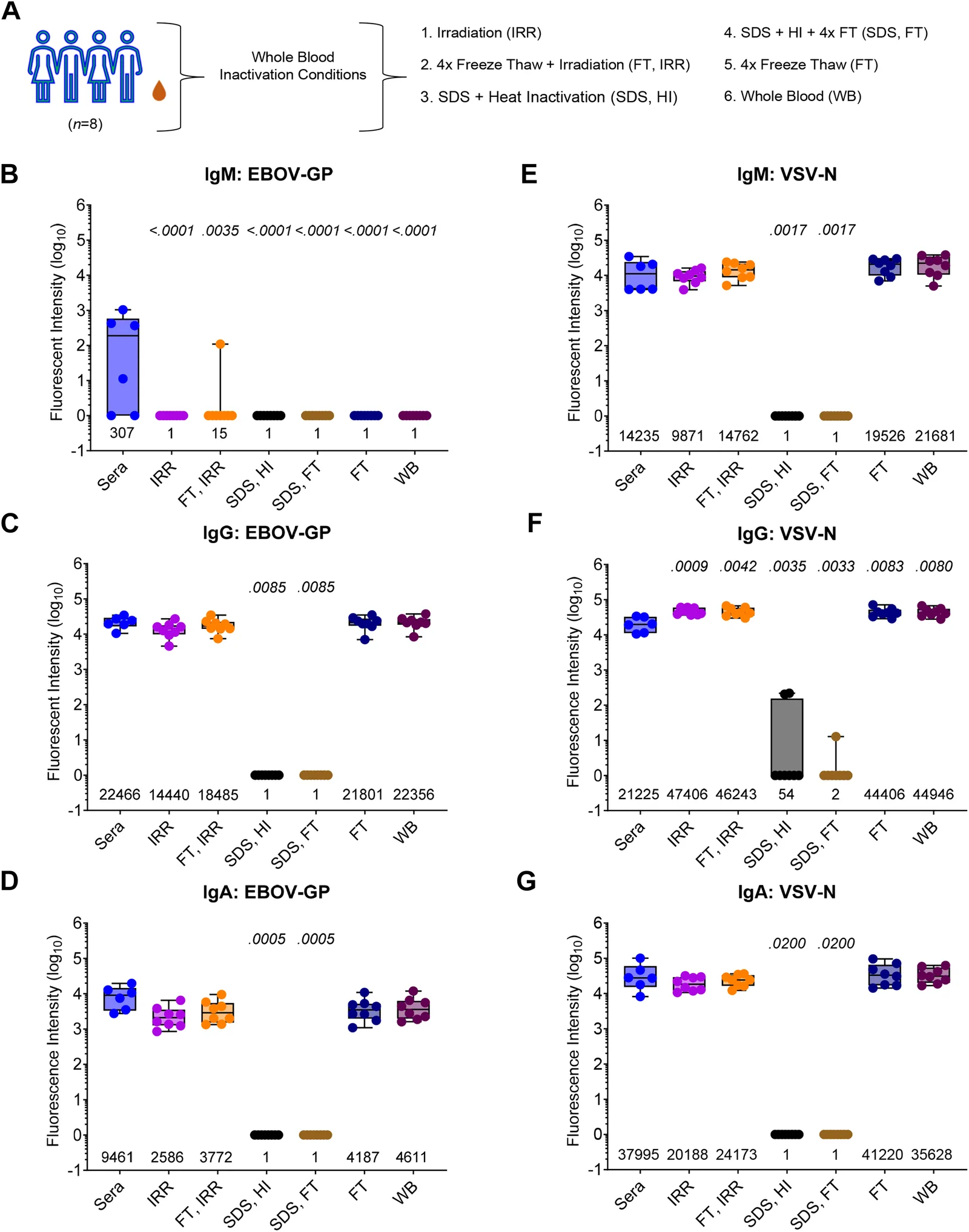
**Whole blood freeze-thawing or gamma irradiation with or without freeze-thawing are optimal approaches for detection of rVSVΔG-ZEBOV-GP vaccine-induced antibodies using the multiplex assay.** Whole blood was collected from eight people who had been vaccinated with rVSVΔG-ZEBOV-GP 5–6 years before. (A) Whole blood (WB) was separated into groups for treatment by six different methods: irradiation (IRR), freeze thaw (FT) four times + IRR, sodium dodecyl sulphate (SDS) + heat inactivation (HI), and SDS treatment + HI + FT, FT four times, as well as serum extraction (as a control group). All samples were evaluated for antibody reactivity against Ebola virus (EBOV) glycoprotein (GP) (B–D) and vesicular stomatitis Indiana virus (VSV) nucleoprotein (E–G), with IgM (B and E), IgG (C and F), and IgA (D and G) isotypes. Mean fluorescent intensity (MFI) values for each handling condition is shown above bars. Differences between untreated whole blood (WB) and each handling condition were examined for statistical significance by paired *t*-test, and only statistically significant values (*p*-values <0.05) are shown.

## Discussion

Identifying accurate early and long-lasting signatures of rVSVΔG-ZEBOV-GP vaccination is challenging with traditional serologic assays: the detection of anti-EBOV-GP IgG does not distinguish between natural EBOV infection and vaccination. These limitations hinder investigation of the real-world effectiveness of the vaccine, the prevalence and consequences of breakthrough EBOV infections, and epidemiological surveillance in an increasingly vaccinated at-risk population. Here, we use a multiplex assay, notably including the evaluation of IgM, IgG, and IgA antibody responses against both EBOV-GP and VSV-N antigens, to provide a comprehensive view of early and longitudinal humoural immune response kinetics to vaccination, identifying anti-VSV-N IgA as an early and durable biomarker of vaccine receipt.

Observed antibody response kinetics align with those previously characterised after rVSVΔG-ZEBOV-GP vaccination, including the rapid induction of anti-EBOV-GP humoural responses^3^^,^^21^^,^^24^ that likely underlie vaccine efficacy^3^ and real-world effectiveness.^4^ Data on the detection of VSV-specific immune responses in the context of vaccination are limited. In US or European subjects of phase I clinical trials, only small or transient antibody responses to VSV matrix protein or VSV-N were detected.^25^^,^^26^ In African subjects over longer time periods post-vaccination, the detection of anti-VSV-N IgG identified rVSVΔG-ZEBOV-GP-vaccinated individuals, and was correlated with anti-EBOV-GP IgG, but via a limited throughput singleplex assay that targeted detection of anti-VSV IgG only.^27^ More recently, a four-antigen IgG assay (incorporating EBOV GP, a modified VSV N, and EBOV secreted glycoprotein [sGP]) identified a signature of rVSVΔG-ZEBOV-GP vaccination at later time-points post-vaccination, but did not evaluate early timepoints (assessed only at 6 and 12 months after vaccination), and did not evaluate other antibody isotypes^20^ The Ebola-Detect 2.0 multiplex assay adds precision, revealing differences in the timing and magnitude of IgM, IgG, and IgA responses to both EBOV-GP and to the VSV-N antigens. Indeed, the kinetics of antibody responses were quantitatively and qualitatively different between the EBOV-GP and VSV-N antigens, both in terms of absolute antibody levels, isotypes, emergence, and durability. Whether or not the inclusion of antibodies to EBOV-sGP could further enhance performance, is of interest. Importantly, anti-VSV-N antibodies identify individuals who received the rVSVΔG-ZEBOV-GP vaccine but do not constitute a direct surrogate of EBOV-specific protective immunity, nor do they distinguish between vaccinated individuals and those who may have also experienced natural EBOV infection. Concurrent detection of anti-EBOV-GP antibodies and, ideally, antibodies to EBOV antigens absent from the vaccine (e.g., VP40) would be required to draw conclusions about EBOV infection history, as was eluded using differential serodiagnostic assay in our previous study.^19^ This distinction should be clearly communicated when this assay is used in outbreak settings.

It is a fundamental immunological principle that antigen delivered by intramuscular (IM) vaccination drains to regional lymph nodes where B-cell activation is biased toward IgG-dominated class-switching and dominant IgG responses in the blood, at least to the vaccine target antigen.^28^ In the present study, the early detection of high levels of anti-VSV-N IgA soon (versus IgM) and durably (versus IgG) after IM vaccination is of interest. IgA antibodies after IM vaccination have been reported in the context of vaccines to prevent COVID-19^29^^,^^30^ and EVD.^15^^,^^31^ Limited investigation of anti-EBOV-GP IgA responses in rVSVΔG-ZEBOV-GP-vaccinated humans suggests development of anti-EBOV-GP IgA was clearly subordinate to IgG^15^; however, anti-VSV-N IgA responses have not been studied in humans. Whether the early and sustained anti-VSV-N IgA vector responses observed here reflect unique distribution of the replicating vector or unique responses in these vaccinees (e.g., host genetics, different prior exposures to “VSV-like” viruses) is uncertain, and needs further evaluation. Regardless, the early detection of anti-VSV-N IgA, combined with the high sensitivity and specificity of the assay, suggest added value in a significantly earlier detection window after vaccination. For example, in ring vaccination studies, the detection of anti-VSV-N IgA by day 10 could identify vaccine ‘takers’ vs ‘non-takers’ and thus serve as a non-mechanistic correlate of protection (COP) for disease diagnosed after day 10. Notably, detection of anti-VSV-N IgA primarily biomarks vaccination status and should not be considered a mechanistic COP. The simultaneous multiplex measurement of anti-EBOV-GP IgG (the mechanistically plausible COP) acts as a necessary failsafe to ensure that functional humoural immunity is adequately evaluated.

Over the longer-term, the sustained detection of anti-VSV-N IgA and IgG up to 5–6 years in whole blood after vaccination suggest vector-specific antibodies may be a good serological marker of vaccination and provide insight into the durability of vaccine-induced immunity against VSV and EBOV, both of which may contribute to evaluation of the overall effectiveness of vaccination programs.^32^^,^^33^ The more comprehensive quantification and dynamics of IgM, IgG, and IgA responses could also provide insight into whether and how different antibody isotypes contribute to protection against EBOV infection, amelioration of disease progression, and in identifying correlates of protection.^19^

The compatibility of gamma-irradiation and freeze-thaw cycles (which reflects sample handling and stability) with the robust antibody detection capability of the multiplex assay is significant for future feasibility in diverse research contexts, not only in maximum-containment laboratories but also in field settings, in which whole blood samples must often be stored for future testing.^34^^,^^35^ The unusually high persistence of VSV-N IgM after 5–6 years is curious and may reflect long-lived plasma cells or persistent antigen stimulation, warranting further investigation.

This study has several limitations. First, diagnostic performance (sensitivity and specificity) requires further validation in independent cohorts; the reported sensitivity and specificity values may be optimistic, as threshold values were derived from the same dataset used to evaluate assay performance, and independent validation in a separate cohort would be necessary. Second, aberrant immune responses in immunocompromised hosts, such as those with malnutrition or HIV-1 infection, prevalent in some EVD-at risk areas, may complicate assay interpretation by altering the expected kinetics or magnitude of the vaccine-induced response. As our study was mostly performed in 68 healthy adults from the US and Liberia, evaluation of the utility of the multiplex assay would ideally be expanded to larger cohorts from at-risk areas in Africa.

Our work sets the stage to address a critical question of the overall real-world protection afforded by rVSVΔG-ZEBOV-GP vaccination. Combined detection of antibodies to the vaccine vector backbone and to multiple Ebola virus antigens (e.g., EBOV matrix protein [VP40]) that are not present in the vaccine nor cross-reactive with long-lived mAb-based therapeutics might enable identification of “true” EBOV infection even in vaccinated individuals and those who may have received various pre- or post-exposure prophylactic interventions.^19^ As such, a multi-EBOV-antigen assay would minimise misclassification of vaccinated individuals (with anti-EBOV GP seropositivity) as EVD survivors, and minimise the associated social stigma. In the context of “true” breakthrough infections, this assay could distinguish immunised and vaccine-naive individuals. rVSV-based vaccines have been developed or are under study for other filovirus diseases (e.g., Sudan virus disease [SVD]^36^^,^^37^ and BVD^38^) as well as other high-consequence viral infections (e.g., Lassa fever^39^). The geographic overlap among high-risk areas for these infections, as well as the increasing numbers of individuals that will have been previously vaccinated (regardless of vaccine antigen), and the likely introduction of rVSVΔG-ZEBOV-GP-boosting strategies,^15^ argue that accurate determination of vaccine status is likely to become even more important but also more complex over time. In this regard, the assay approach developed here can be used and/or adapted for purpose to study immune responses to other emerging viruses, contributing to a more rapid and comprehensive approach to vaccine development and epidemic response,^40^ and enhancing global preparedness for future outbreaks.^41^

In contrast to our prior work targeting the ELISA-based detection of only IgG (Ebola-Detect-1.0),^19^ the detection of IgM and IgA antibodies and the inclusion of VSV-N antigen enable confirmation of vaccination status with more comprehensive antibody isotypes against VSV-N and EBOV antigens. Early detection of vaccination status during outbreaks is critical for identifying immunity gaps, distinguishing naturally infected individuals from vaccinated ones, and evaluating vaccine effectiveness in real-time. IgA antibodies are particularly valuable because they appeared rapidly, often within days, providing markers of vaccination induced immunity. Multiplex assays like Ebola-Detect 2.0 that simultaneously detect IgA against the vaccine vector (VSV–N) and indicators of natural infection (EBOV antigens) could enable rapid differentiation between vaccinated and naturally infected individuals and provide practicable data essential for outbreak response, the evaluation of vaccination campaigns, and broader epidemiological surveillance.

In the absence of approved vaccines for the BVD outbreak, whether the potential cross-protection afforded by rVSV-vectored EVD and/or SVD vaccines in nonhuman primate models^6^ is meaningful in humans is an open question. The current BVD outbreak overlaps significantly with multiple prior EVD outbreaks in which the rVSV-ZEBOV-GP (in Democratic Republic of the Congo) or Sudan virus-targeted rVSV-SUDV-GP (in Uganda) vaccines have been used, particularly in at-risk health-care workers. Accurate identification of pre-existing rVSV-vectored immunity to both vector- and target-antigen will be useful in an increasingly complex vaccine landscape to clarify these complex interactions and inform appropriate public health measures.

## Contributors

S.K, D.A.F, and I.M.B. contributed to the design of the study.

S.K., I.M.B., and D.A.F. contributed to study oversight.

S.K. and D.A.F. contributed to data collection and data cleaning.

O.P., V.C., and S.K. conducted statistical analyses and had full access to and verified all the data. O.P., S.K., and K.H. performed research.

S.K., O.P., and D.A.F. interpreted the data and results.

S.K. drafted the manuscript with contributions from O.P., I.M.B., V.C., J.H.K., S.K., M.K., S.M.B, O.T.M., I.C., L.H., R.T.D., H.C.L., D.A.F., and S.K.

All authors confirm that they had full access to all the data in the study and accept responsibility to submit for publication. All authors read and approved the final version of the manuscript. S.K. and D.A.F. verify the underlying data of the study.

## Data sharing statement

All data needed to evaluate the conclusions in the paper are provided in the manuscript.

## Code sharing

No custom codes were used in this study.

## Declaration of interests

The authors declare that no conflicts of interest exist.
