# Supplementary material for: Early detection of immunoglobulin A against a vaccine backbone protein enables fast, durable, and objective identification of individuals vaccinated against Ebola virus
Source: eBioMedicine. 2026 Oct 16;133:106515. doi: 10.1016/j.ebiom.2026.106515 (PMC13696117; doi:10.1016/j.ebiom.2026.106515)
Supplement: Supplementary file 1 [file mmc1.pdf]

Supplementary Table 1: Supportive data for Figure 3 for the 95% CIs and standard error for sensitivity, specificity and AUC

		EBOV-GP							
Isotype	Figure panel	D0 vs	MFI >	Sensitivity%	Specificity%	AUC	*p*	95% CI	Standard Error
IgM:	A	D7	375.3	3.3	100	0.5	*0.9941*	.3533-.6479	0.07516
		D28	17.4	80	96.7	0.89	<*.0001*	.805-.984	0.0459
		D180	8	23.3	96.7	0.6	*0.1809*	.4563-.7448	0.07359
		D360	348.8	10	100	0.54	*0.6414*	.3880-.6820	0.07498
IgG:	B	D7	113	40	80	0.51	*0.8476*	*.3661-.6628*	0.07571
		D28	1401	93.3	93.3	0.98	<*.0001*	*.958-1*	0.0123
		D180	1194	83.3	93.3	0.96	<*.0001*	*.9172-1*	0.02128
		D360	1481	86.7	93.3	0.97	<*.0001*	*.9402-1*	0.01633
IgA:	C	D7	10.6	30	83.3	0.57	*0.3632*	*.4225-.7142*	0.07442
		D28	299	93.3	93.3	0.97	<*.0001*	*.92-1*	0.02435
		D180	96.4	90	90	0.9	<*.0001*	*.8158-.9898*	0.04438
		D360	46.1	86.7	86.7	0.88	<*.0001*	*.7845-.9755*	0.04873
VSV-N									
Isotype		D0 vs	MFI >	Sensitivity%	Specificity%	AUC	*p*	95% CI	Standard Error
IgM:	D	D7	271.8	90	80	0.88	*0.0001*	*.7908-.9781*	0.04777
		D28	4244	96.7	93.3	0.96	*0.0001*	*.9077-1*	0.02667
		D180	385.8	93.3	83.3	0.89	*0.0001*	*.8029-.9860*	0.04669
		D360	6.5	83.3	73.3	0.8	*0.0001*	*.6863-.9203*	0.05970
IgG:	E	D7	371.8	93.3	73.3	0.83	*0.0001*	*.7216-.9439*	0.05671
		D28	1876	96.7	93.3	0.97	*0.0001*	*.9405-1*	0.01734
		D180	1968	96.7	93.3	0.99	*0.0001*	*.968-1*	0.009807
		D360	2678	96.7	93.3	0.99	*0.0001*	*.9641-1*	0.01092
IgA:	F	D7	1164	96.7	93.3	0.97	*0.0001*	*.9349-1*	0.01960
		D28	5851	100	96.7	0.99	*0.0001*	*.9853-1*	0.005245
		D180	4083	96.7	96.7	0.99	*0.0001*	*.9646-1*	0.01183
		D360	4546	96.7	96.7	0.99	*0.0001*	*.9615-1*	0.01286

Supplementary Figure S1: rVSVΔG-ZEBOV-GP vaccination results in anti-vaccine backbone immunoglobulin A responses that precede anti-vaccine target antigen responses in the multiplex assay. IgM/IgG/IgA reactivity in longitudinally collected human serum samples with Ebola virus (EBOV) glycoprotein (GP) and vesicular stomatitis Indiana virus (VSV) nucleoprotein (N) after rVSVΔG-ZEBOV-GP vaccination. Fluorescence intensity indicating antibody levels of a 100-fold serum dilution for every participant at each timepoint from 60 adults against EBOV-GP and VSV-N. Results for the placebo group (n=30, days 0 and 28 only) are shown in black, whereas rVSVΔG-ZEBOV-GP vaccinated group (n=30 for days 0, 7, 28, 180, 360; n=4 for days 3, 10, 14) binding antibodies against EBOV-GP (A–C; in green) and VSV-N (D–F; in blue) are shown with IgM (A and D), IgG (B and E) and IgA (C and F), respectively. Mean fluorescent intensity values for each timepoint are shown. Some vaccinated participants have MFI value of 1 on multiple time-points, and therefore such vaccinees individual trendlines are not distinguishable. Differences between time-points within each group were examined for statistical significance by paired Student's t-test, and statistically significant values (*p*-values <0.05) are shown.

Figure S1

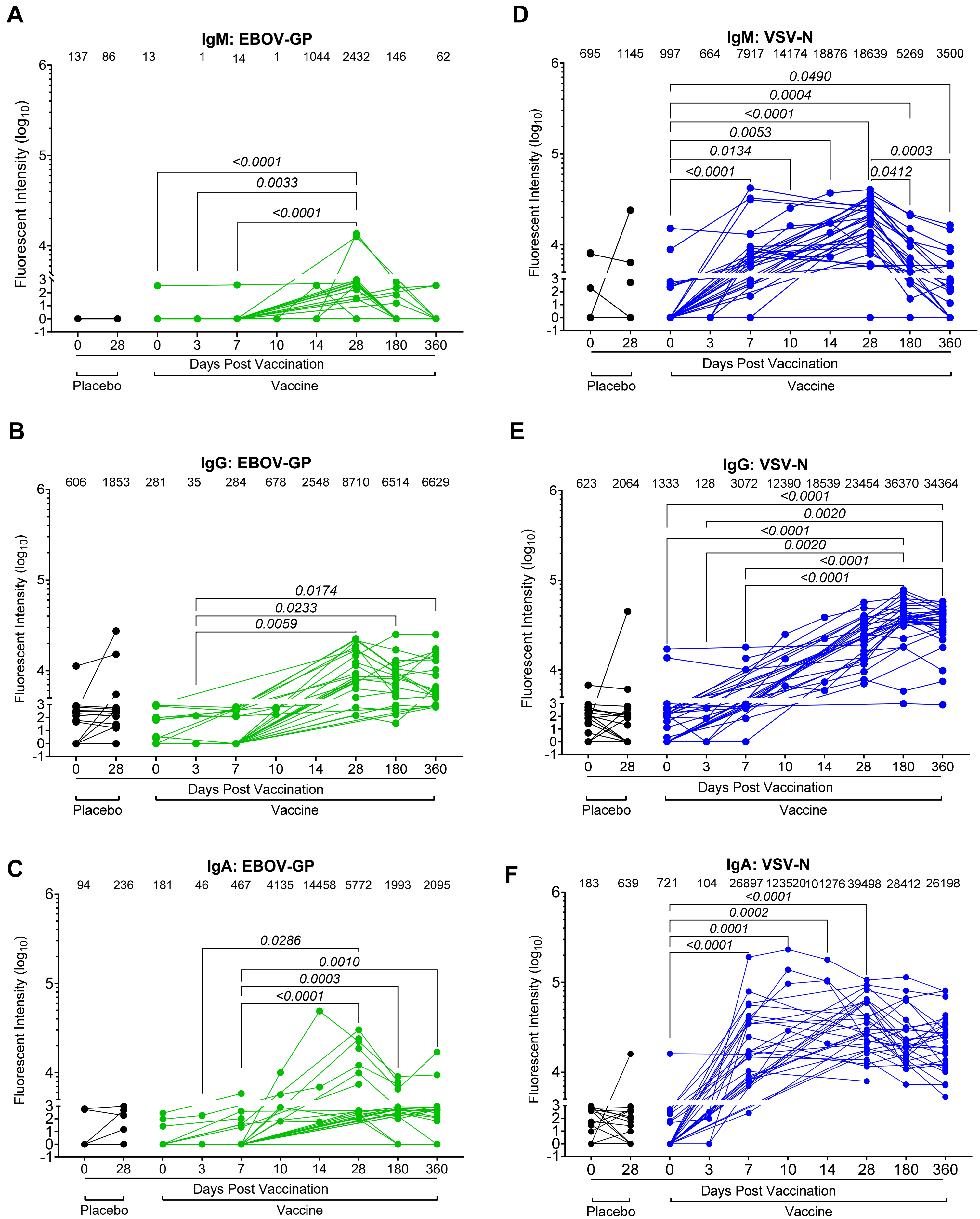
A
IgM: EBOV-GP
137 86 13 1 14 1 1044 2432 146 62
<0.0001
0.0033
<0.0001
Fluorescent Intensity (log10)
Days Post Vaccination
Placebo
Vaccine
B
IgG: EBOV-GP
606 1853 281 35 284 678 2548 8710 6514 6629
0.0174
0.0233
0.0059
Fluorescent Intensity (log10)
Days Post Vaccination
Placebo
Vaccine
C
IgA: EBOV-GP
94 236 181 46 467 4135 14458 5772 1993 2095
0.0286
0.0010
0.0003
<0.0001
Fluorescent Intensity (log10)
Days Post Vaccination
Placebo
Vaccine
D
IgM: VSV-N
695 1145 997 664 7917 14174 18876 18639 5269 3500
0.0490
0.0004
<0.0001
0.0053
0.0134
<0.0001
0.0003
0.0412
Fluorescent Intensity (log10)
Days Post Vaccination
Placebo
Vaccine
E
IgG: VSV-N
623 2064 1333 128 3072 12390 18539 23454 36370 34364
<0.0001
0.0020
<0.0001
0.0020
<0.0001
<0.0001
Fluorescent Intensity (log10)
Days Post Vaccination
Placebo
Vaccine
F
IgA: VSV-N
183 639 721 104 26897 123520101276 39498 28412 26198
<0.0001
0.0002
0.0001
<0.0001
Fluorescent Intensity (log10)
Days Post Vaccination
Placebo
Vaccine
